# Chemometric Evaluation of RI-Induced Phytochemicals in *Phaseolus vulgaris* Seeds Indicate an Improvement on Liver Enzymes in Obese Rats

**DOI:** 10.3390/molecules28247983

**Published:** 2023-12-07

**Authors:** Mayra Denise Herrera, Iza Fernanda Pérez-Ramírez, Rosalía Reynoso-Camacho, Luis Roberto Reveles-Torres, Miguel Servín-Palestina, Angelica Judith Granados-López, Claudia Araceli Reyes-Estrada, Jesús Adrián López

**Affiliations:** 1 Campo Experimental Zacatecas (CEZAC-INIFAP), Carretera Zacatecas-Fresnillo Km 24.5, Calera de VR, Zacatecas 98500, Mexico; mayradherrera@gmail.com (M.D.H.); lreveles@gmail.com (L.R.R.-T.); servin.miguel@inifap.gob.mx (M.S.-P.); 2 Unidad Académica de Ciencias Biológicas, Universidad Autónoma de Zacatecas “Francisco García Salinas”, Avenida Preparatoria No. 301, Colonia Hidráulica, Zacatecas 98068, Mexico; agranados@uaz.edu.mx; 3 Research and Graduate Studies in Food Science, Faculty of Chemistry, Autonomous University of Queretaro, Queretaro 76010, Mexico; iza.perez@uaq.mx (I.F.P.-R.); rrcamachomx@yahoo.com.mx (R.R.-C.); 4 Unidad Académica de Ciencias Químicas, Universidad Autónoma de Zacatecas, Campus Siglo XXI, Villanueva–Zacatecas, La Escondida, Zacatecas 98160, Mexico

**Keywords:** chemometric, restriction irrigation, *Phaseolus vulgaris*

## Abstract

Liver enzymes alterations (activity or quantity increase) have been recognized as biomarkers of obesity-related abnormal liver function. The intake of healthy foods can improve the activity of enzymes like aspartate and alanine aminotransferases (AST, ALT), γ-glutaminyl transferase (GGT), and alkaline phosphatase (ALP). Beans have a high concentration of several phytochemicals; however, Restriction Irrigation (RI) during plant development amends their synthesis. Using chemometric tools, we evaluated the capacity of RI-induced phytochemicals to ameliorate the high activity of liver enzymes in obese rats. The rats were induced with a high-fat diet for 4 months, subsequently fed with 20% cooked beans from well-watered plants (100/100), or from plants subjected to RI at the vegetative or reproduction stage (50/100, 100/50), or during the whole cycle (50/50) for 3 months. A partial least square discriminant analysis indicated that mostly flavonols have a significant association with serum AST and ALT activity, while isoflavones lowered GGT and ALP. For AST and ALT activity in the liver, saponins remained significant for hepatocellular protection and flavonoids remained significant as hepatobiliary protectants by lowering GGT and ALP. A principal component analysis demonstrated that several flavonoids differentiated 100/50 treatment from the rest, while some saponins were correlated to 50/100 and 50/50 treatments. The intake of beans cultivated under RI improves obesity-impaired liver alterations.

## 1. Introduction

Obesity is a metabolic disorder characterized by an imbalance between fat intake and energy expenditure, and it is a well-known risk factor for metabolic syndrome, which can further lead to diabetes, cardiovascular diseases, and non-alcoholic fatty liver disease (NAFLD) causing abnormal liver functions [1,2]. Some studies have shown that liver enzymes, including alanine aminotransferase (ALT), aspartate aminotransferase (AST), γ-glutamyl transferase (GGT), and alkaline phosphatase (ALP), are valid indicators of liver injury strongly associated to the hepatic accumulation of lipids and increased oxidative stress [2,3,4]. Hence, pharmacological agents have been developed as a strategy to aid weight loss alongside lifestyle interventions; however, several pharmacological treatments have been withdrawn from the market due to side and/or adverse effects [5]. Therefore, finding natural products to ameliorate obesity and its complications is highly recommended. The common bean (*Phaseolus vulgaris*), along with other pulses, has extensively been studied as an antiobesogenic food [6]. Currently, the common bean is the most important grain legume for human-direct consumption, contributing more than 8700 hg/ha to global legume production [7]. Its functional potential has been related to its content of phytochemicals, synthetized from the primary and secondary metabolism which are dependent on the biotic and abiotic factors acting during plant growth [8]. Based on a previous chemometric approach performed among the phytochemicals concentration and digestive enzyme inhibition capacity of the Pinto Saltillo and Raramuri common bean cultivars, affected by different soil water supply regimes, the restricted irrigation (RI) was identified as an agricultural production strategy to improve the functional properties of the cooked beans [9] Likewise, the phytochemical fingerprint of the Pinto Durango bean seeds was previously obtained. Several individual flavonoids, phenolic acids, saponins, phytosterols, and oligosaccharides where identified, and a partial least square-discriminant analysis (PLS-DA) indicated that the seeds from the plants grown under RI during the whole growth cycle were differentiated from those grown under full irrigation and a terminal severe drought due to their high content of quercetin 3-O-rhamnoside and luteolin 7-O-glucoside, whereas seed yield was not penalized [10]. Thus, the use of the RI strategy might induce the biosynthesis of the phytochemicals with the capacity to alleviate obesity related liver disorders, such as increased liver enzymes activity linked to NAFLD, and other obesity-linked outcomes, including diabetes, hypertension, dyslipidemia, metabolic syndrome, and cardiovascular diseases [11]. Therefore, this study aimed to use chemometric tools to identify the RI-induced phytochemicals of cooked Dalia common bean seeds with a potential to improve the ALT, AST, GGT, and ALP liver enzymes alterations in treated obese-induced rats.

## 2. Results and Discussion

### 2.1. Phytochemical Profile of Cooked Dalia Bean Grown under Different Irrigation Regimes

Treatment 100/50 induced a higher concentration of more individual flavonoids than the remaining irrigation treatments (Table 1). The concentrations of the flavanols (−)-epicatechin hexoside and (+)-catechin hexoside were 2.1 and 1.6 times higher, respectively, than that obtained from the seeds harvested from the plants that were grown under full irrigation (100/100). Meanwhile, although the amount of kaempferol was higher in the seeds from the plants subjected to full irrigation, some derivatives like -pentosyl-hexoside, -hexoside, -acetyl-hexoside and, -malonyl-hexoside were 1.2, 2.2, 2.7, and 2.1 times higher, respectively, in the seeds from the 100/50-treated plants. The latter also led to higher concentrations of luteolin and luteolin apiosyl-hexoside (2-fold), daidzein (1.3-fold), daidzein hexoside (1.6-fold), genistein (1.2-fold) and trihydroxyisoflavan (1.3-fold). Meanwhile, the seeds obtained by the 50/100 treatment had only a statistically higher concentration of naringenin, whereas the 50/50 did not significantly increase any identified flavonoids.

According to Farouk and Amani [12] and Pérez-Álvarez et al. [13], a drought prompts oxidative stress in plants and stimulates various biological changes, leading to the production of a reactive oxygen species (ROS), which provokes damage to the cell and tissues which increases the production of secondary metabolites, such as polyphenols. Moreover, flavonoids are reported to be involved in the resilience of stress caused by an inadequate water supply by scavenging free radicals via a donation of an electron or hydrogen to the ROS [8]. Variations in their accumulation will preferably be among compounds with greater antioxidant power, such as flavonols and flavones [14]. For instance, Monika and Yadav [15] found that flavanols such as epicatechin regulate antioxidant enzymes to promote the cell detoxification mechanism against the ROS. Additionally, Zarrouk et al. [16] reported that stress promotes the reduction in the UPD-glucose-flavonoid 3-O-glucosyltransferase activity, increasing the flavonol content. Moreover, the water status in plants is directly correlated with changes in the stomatal conductance, affecting photosynthesis, which is inhibited by decreasing intercellular CO_2_ concentrations depending on the stomatal closure. Due to the highly energetic reactions of photosynthesis, the reduction of molecular oxygen generates a toxic ROS, also alleviated by flavanones like naringenin [17], which can be associated with the current results.

Only three hydroxybenzoic acids and one hydroxycinnamic acid were identified. The concentrations of vanillic and dihydroxybenzoic acids were statistically higher in the beans from plants grown under 100/50 (1.6 and 1.4 times higher than 100/100), whereas the 50/100 water supply treatment led to the highest concentration of ferulic acid, with a 1.6–fold increase. In the metabolic profiling of leaves from a drought-tolerant genotype of *Arachis hypogaea* L., higher concentrations of vanillic and ferulic acids due to the stimuli of water deprivation meant that the phenolic acids provide tolerance to stress because they release hydroxyl atoms and oxidize themselves, acting as antioxidants [18].

We previously reported that if RI has a place during the whole cycle of the Pinto Durango bean plants, they exhibit a higher accumulation of raffinose, stachyose, and verbascose in the seed; these changes were attributed to the oligosaccharides’ scavenging mechanisms affecting the ROS signaling pathways [10]. However, although the raffinose family oligosaccharides (RFO) were identified in the cooked Dalia beans, no statistical differences were observed (Table 1). This suggests that the activation of galactinol synthase, a key enzyme in the synthesis of RFO [19], is differentiated by the genotype of the cultivar and, more specifically, by their behavior to water stress considering Pinto Durango is categorized as a drought-resistant cultivar, unlike Dalia. This idea also supports our findings on the saponin concentration. The current results exhibit that for Dalia beans, only the soyasaponin ag was affected by withholding water during the plants’ growth, and that the Pinto Durango exhibited a statistically higher concentration of several saponins; however, the soyasaponin ag was identified as a variable of importance [10]. As plant steroids are capable of increasing tolerance to drought by controlling the opening of the stomata, the soyasaponin ag may be of importance to escape oxidative stress, either to drought-resistant or non-resistant cultivars.

### 2.2. Effect of Cooked Dalia Bean Intake on Liver Enzymes

**Figure 1:**
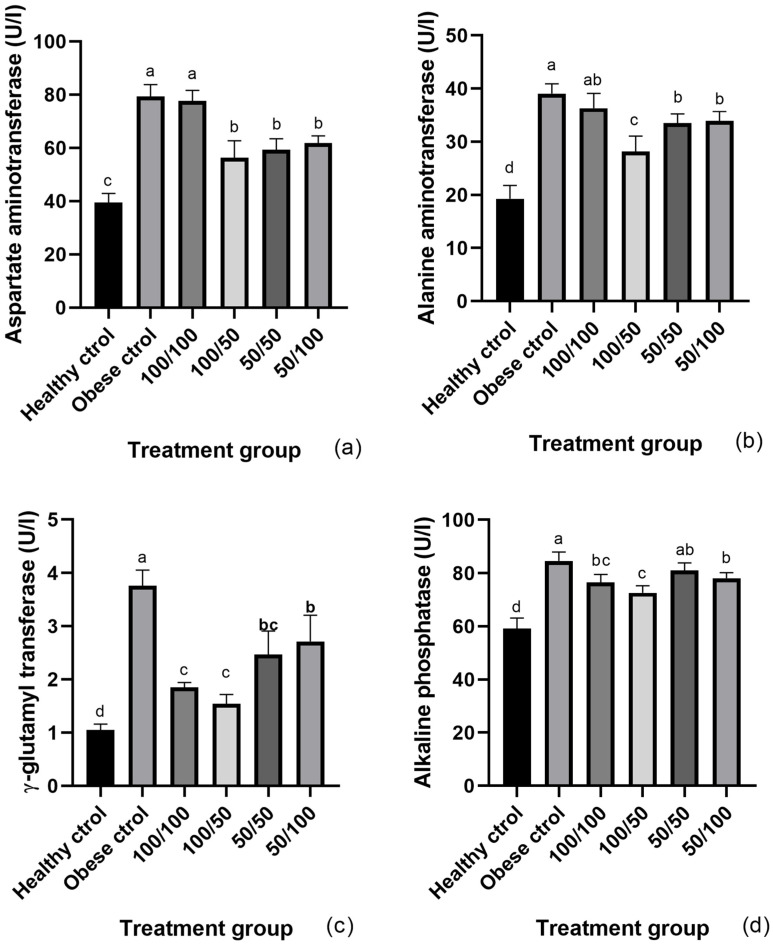
Serum AST (**a**), ALT (**b**), GGT (**c**), and ALT (**d**). Values are expressed as mean ± SEM. Different letter above bars represent statistical difference (*p* < 0.05) with the Tukey test. For AST, ALT, GGA, and FA, *p* < 0.0001.

Apart from other biochemical factors, liver enzymes activities have also been reported to increase with obesity [1]. The serum levels of AST, ALT, GGT, and ALP activity are shown in Figure 1. The rats from the obese control had a 2.0–fold increase in AST activity, in comparison to the healthy control. No statistical difference was found between the obese control and the 100/100-treated group (Figure 1a). The obese control also showed a 2.0-fold increase in the serum ALT in comparison to the healthy control. Although treatments 50/50 and 50/100 lowered the activity of this enzyme, the outstanding water regime treatment was 100/50, which decreased the ALT activity 0.7 times (Figure 1b). Obesity had a significant association with a greater risk of elevated ALT and AST [2]. Additionally, obesity-related hepatic steatosis has been associated with decreased liver function by elevating the plasma activity of ALT and/or AST and is considered a marker of hepatocellular damage [20]. Nagell et al. [21] mentioned that the elevated serum levels of aminotransferases are not due to an increased enzyme synthesis but rather the manifestation of an enhanced enzyme release caused by liver cell death or liver injury with an increase in permeability of the hepatocellular membrane.

The highest difference in the liver enzymes between both control groups was observed in their GGT activity. More specifically, the obese control exhibited a 3.8–fold increase. Interestingly, the animals who consumed the beans from the well-watered plants showed lower GGT activity, similar to that observed by the 100/50 group, with a 0.5 and 0.4 times decrease, respectively, in comparison to the obese control (Figure 1c). Several studies have shown a close association between GGT and insulin resistance in the setting of obesity, diabetes, and NAFLD. We previously reported that animals who consumed cooked Dalia from 100/50-treated plants lowered the risk of insulin resistance [22] GGT is a ubiquitous cell surface enzyme that cleaves glutathione. Therefore, there is strong evidence of an association between its elevated activity and increased oxidative stress, thus glutathione, the intracellular synthesis of which is dependent on GGT [4]. Consequently, an inverse association between GGT activity and circulating antioxidants is expected which is in agreement with the higher concentration of metabolites capable of counteracting the ROS induced by the 100/50 irrigation treatment.

The activity of the serum ALP was higher in the obese animals (1.4-fold). Although 100/100 and 50/100 treated groups had a significant decrease, the greatest diminution was observed when the animals consumed beans from the 100/50-treated plants (Figure 1d). ALP has also been reported to increase obesity by its release into the blood circulation in excessive amounts where its activity is enhanced and anticipates disproportionate intracellular fat depots [1].

**Figure 2:**
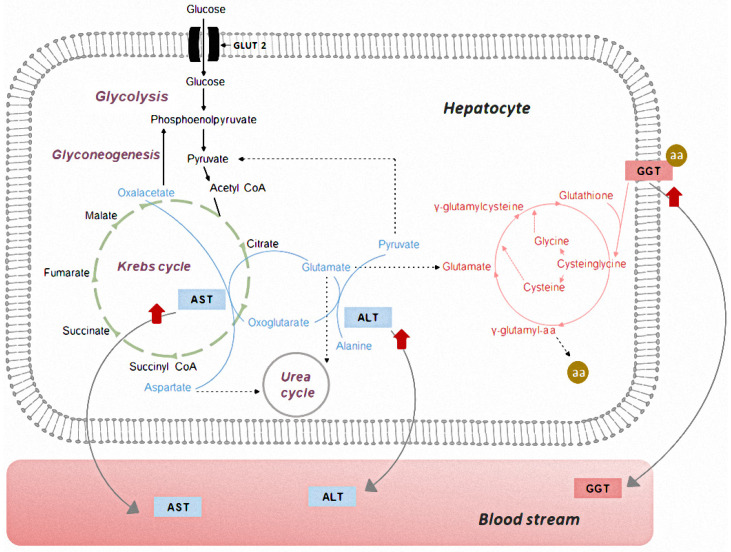
Simplified overview of the mechanisms of AST and ALT (in blue) and GGT (in pink). Red arrows indicate a higher release of liver enzymes to blood.

For a better understanding of the current results, Figure 2 depicts the reactions in which the liver enzymes are involved. Aminotransferases are a group of enzymes that catalyze the interconversion of amino acids and oxoacids by transfer amino groups. AST and ALT are the two aminotransferases of greatest clinical significance. AST catalyzes the reversible reaction of transamination between L-aspartate and oxoglutarate to L-glutamate and oxoalacetatee. AST is also important in the maintenance of a supply of oxaloacetate required for glyconeogenesis [23]. On the other hand, the ALT enzyme catalyzes the transfer of amino groups from the L-alanine to oxoglutarate, and the conversion products are L-glutamate and pyruvate. The process is critical in the liver for the Krebs cycle, where pyruvate can then be used to produce cellular energy [24]. However, according to Oh et al. [25] any process that leads to a loss of hepatocyte membrane integrity or hepatocellular necrosis results in the release of AST and ALT in higher concentrations in the serum (red arrows).

GGT and ALP are recognized as serum biomarkers of hepatobiliary injury, especially cholestasis and biliary effects [21,26]. GGT catalyzes the transfer of γ-glutamyl residues between peptides or from one peptide to an L-amino acid and, thereby, plays a physiological role in the transmembrane transport of amino acids in the γ-glutamyl cycle and in the cleavage of glutathione and its conjugates. Increased serum GGT levels (red arrow) in liver disease are due to a cell membrane injury with an enhanced release of the enzyme or to the detachment of a membrane-bound enzyme by the solubilizing action of bile [21]. ALP belongs to an enzyme family that hydrolyzes esters of phosphoric acid in an alkaline milieu. Biliary obstruction increased pressures in the biliary system and elevated concentrations of bile acids, which all stimulate ALP synthesis [27].

Our current results add to the previous findings and suggest that the increase in liver enzymes is associated with health risks, such as obesity. For instance, Choi et al. (2003) [28] found that serum ALT, AST, and GGT activities in overweight men and women were significantly correlated with total body fat; among the subjects, those with fatty liver showed a significantly higher incidence of elevated hepatic enzymes. According to Ndrepepa et al. [4], elevated GGT has been reported to be closely correlated with markers of systemic inflammation which is an important contributor to atherosclerosis and obesity. Additionally, a study conducted in adult Pakistani individuals showed that high activity of ALP during adipogenesis may be an important contributing factor to the development of fat depots in adipose tissue [1]. Recently, in 2021, Liu et al. [2] suggested that obesity is now a significant predictor of abnormal liver test results.

**Figure 3:**
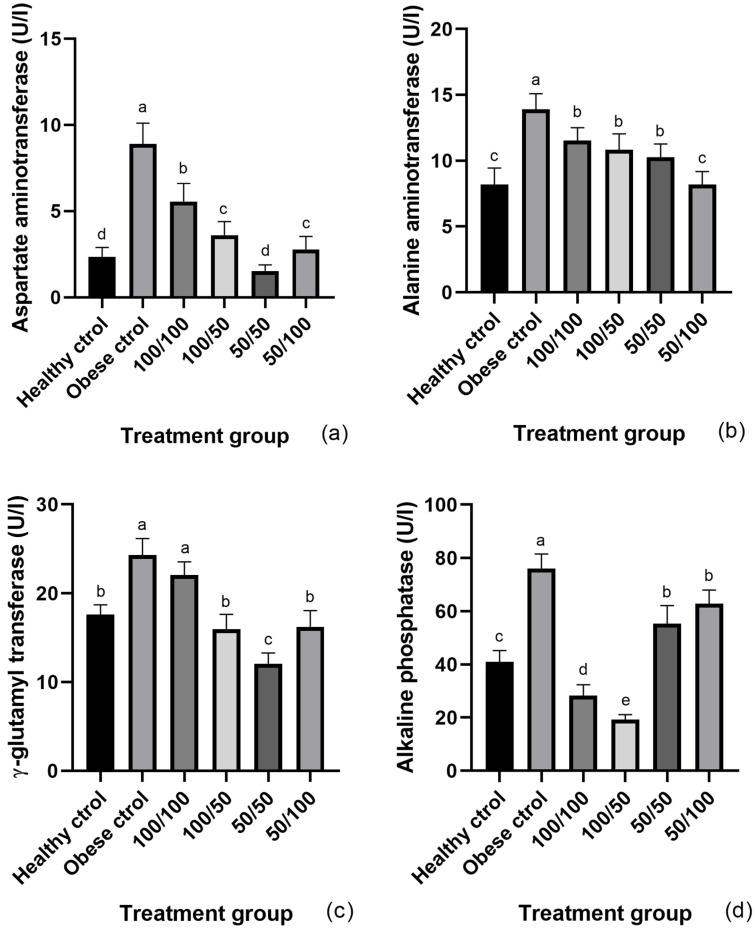
Liver AST (**a**), ALT (**b**), GGT (**c**), and ALT (**d**). Values are expressed as mean ± SEM. Different letter above bars represent statistical difference (*p* < 0.05) with the Tukey test. For AST, GGA, and FA, *p* < 0.0001, for ALT, *p* = 0.0063.

In general, the intake of cooked Dalia beans harvested from the plants grown under RI improved obesity-impaired liver enzymes, evidenced by the measurement of their activity in the serum. These results raised our interest in evaluating the effect of RI on the functionality of the beans by measuring enzyme activity in the liver (Figure 3). As expected, the activity of AST and ALT in obese rats was 3.7- and 1.7-fold (Figure 3a,b) higher, respectively, in comparison to the healthy control. Meanwhile, all bean treatments lowered the activity, with the lowest shown by the animals fed with the 50/50 and 50/100 cooked Dalia beans for AST (0.8 times) and ALT (0.9 times), respectively. In addition, the activity of GGT and ALP was 1.3 (Figure 3c) and 1.9 times (Figure 3d) higher in the liver of the obese rats. Although bean intake reduced both enzymes, the 50/50 excelled by reducing the activity of GGT 0.9 times, and the 100/50 excelled by reducing the activity of ALP 0.5 times, compared to the obese control.

### 2.3. Chemometric Analysis

**Figure 4:**
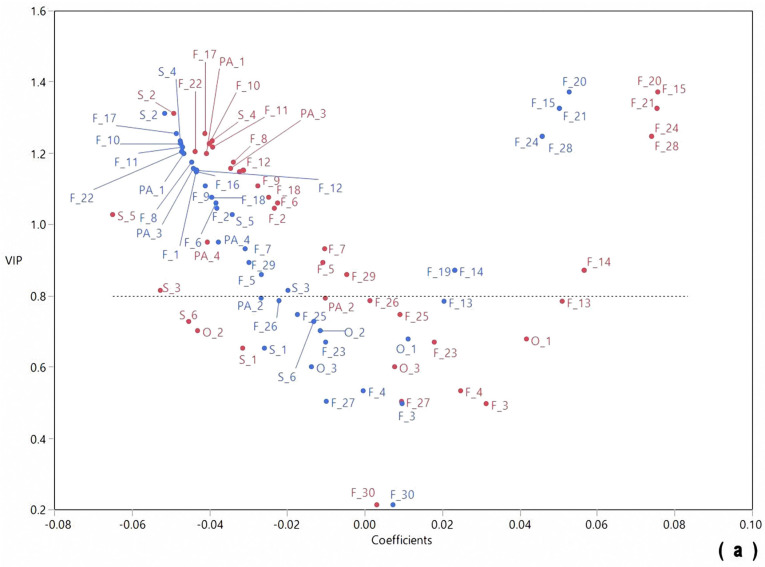

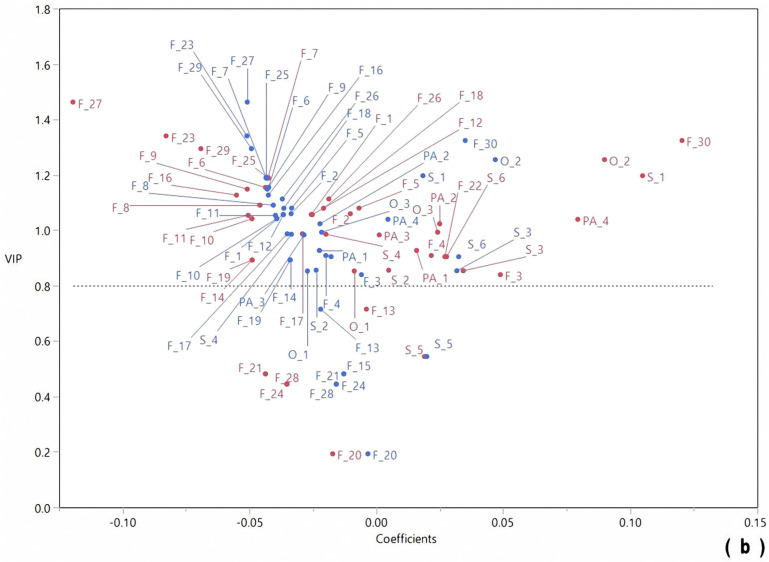
Chemometric analyses using PLS-DA to associate the phytochemical profile of cooked Dalia obtained from plants submitted to different soil moisture treatments with their effect on AST and ALT (**a**) [Red: AST, Blue: ALT], and GGT and ALP (**b**) [Red: GGT, Blue: ALP] in serum of obese-treated rats. The y-axis of PLS-DA graph shows the variable importance of the projection (VIP) score, which summarizes the contribution of the phytochemicals to the model. Phytochemicals with a VIP score > 0.8 have a significant effect. Meanwhile, the x-axis shows coefficients whose magnitude represents the relative importance of each data item towards the separation, which indicates that positive coefficients increase the numerical outcomes of the evaluated phytochemicals, whereas negative coefficients decrease them; therefore, phytochemicals in the upper left quadrant are of major importance.

Spectrometry techniques produce profiles that contain a high amount of information which can profitably be exploited with multivariate mathematic and statistic (chemometric) techniques, such as PCA and PLS-DA [29]. For this study, phytochemicals located in the upper left quadrant of the PLS-DA graphs exerted a beneficial effect on liver enzyme activity considering the phytochemicals with a negative coefficient decreased the outcomes of these parameters. The PLS-DA was performed to identify any association between the phytochemical and the enzymes activity; however, the analysis was separated based on the type of liver injury in which they were involved. Figure 4a represents the relationship between the phytochemicals and the enzymes that are directly linked with hepatocellular damage, while Figure 4b exhibits the association of the enzymes involved in hepatobiliary injury, both in serum samples. Several flavonoids (mostly flavonols), some phenolic acids, and saponins were found to have a significant association with serum AST and ALT activity (Figure 4a). Although most flavonoids associated with an improvement in hepatocellular damage also protect against hepatobiliary injury, isoflavones, such as daidzin, genistein, daidzein, and trihydroxyisoflavan, and the flavone hydroxyluteolin together with raffinose are included as hepatobiliary protectants. Interestingly, no saponins were associated with the lower serum activities of GGT and ALP, while only hydroxybenzoic acid hexoside, among the phenolic acids, had a significant association with both enzymes (Figure 4b).

**Figure 5:**
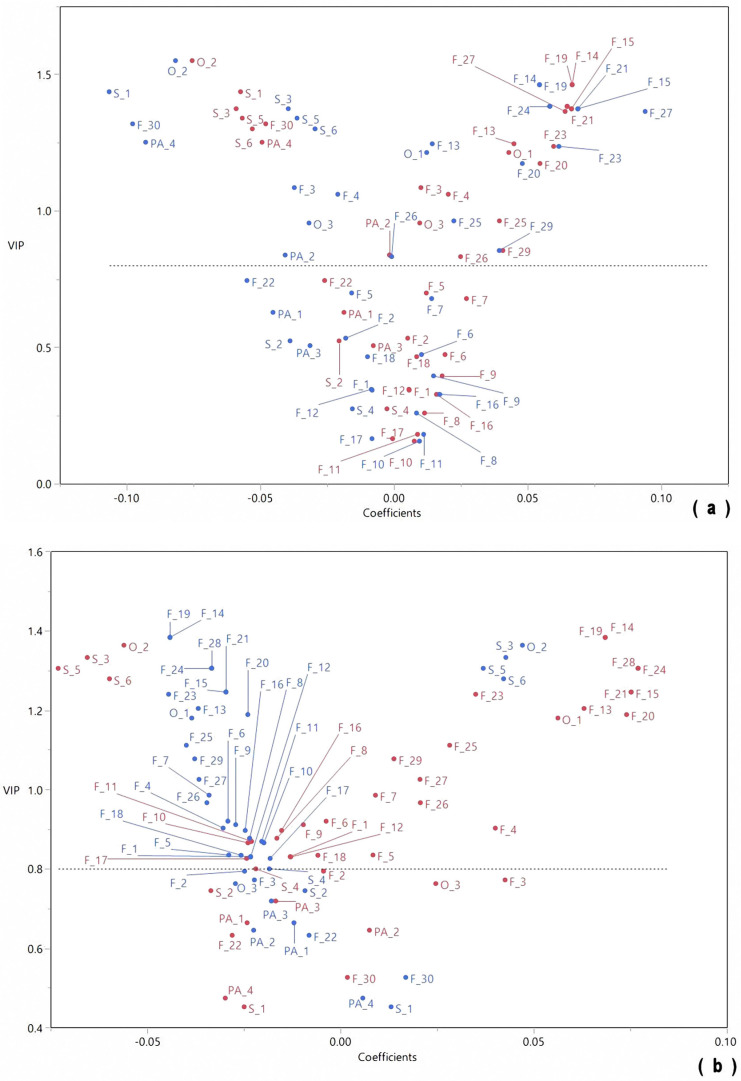
Chemometric analyses using PLS-DA to associate the phytochemical profile of cooked Dalia obtained from plants submitted to different soil moisture treatments with their effect on AST and ALT (**a**) [Red: AST, Blue: ALT] and GGT and ALP (**b**) [Red: GGT, Blue: ALP] in liver of obese-treated rats. The y-axis of PLS-DA graph shows the variable importance of the projection (VIP) score, which summarizes the contribution of the phytochemicals to the model. Phytochemicals with a VIP score > 0.8 have a significant effect. Meanwhile, the x-axis shows coefficients whose magnitude represents the relative importance of each data item towards the separation, which indicates that positive coefficients increase the numerical outcomes of the evaluated phytochemicals, whereas negative coefficients decrease them; therefore, phytochemicals in the upper left quadrant are of major importance.

Conversely, Figure 5a shows that phenolic acids (dihydroxybenzoic and ferulic acids), saponins (soyasaponin Ba (V), ag, bg, and gg), and stachyose played a larger role in the protection against the high activity of AST and ALT in the liver. Only one flavanone (naringenin) was significant for both enzymes. Regarding GGT and ALP activity measured in the liver, the concentrations of the flavonoids were of major importance. The main difference between these enzymes was the association of the saponins and stachyose observed only for the GGT activity and the flavonols, flavones, isoflavones, and raffinose for ALP activity (Figure 5b). Based on the VIP scores (Table S1, Supplementary Material), the outstanding phytochemicals for AST and ALT activity measured in the serum were kaempferol acetyl-hexoside, kaempferol malonyl-hexoside, luteolin, malonylgenistin, and soyasaponin Bb(I), whereas the phytochemicals for GGT and ALP activity measured in the serum were quercetin hexoside-rhamnoside, kaemperol hexoside, daidzin, trihydroxyisoflavan, and naringenin hexoside. The main phytochemicals for the enzyme activity measured in the liver were naringenin and soyasaponins Ba(V), ag, bg, and gg for AST and ALT, and quercetin, (Iso)rhoifolin, tetrahydroxyisoflavanone, eriodictyol, soyasaponin ag, and soyasaponin bg for GGT and ALP. These phytochemicals may have the capacity to prevent or improve liver disease at the hepatocyte damage or hepatobiliary system level. Nonetheless, additional analyses are required, such as molecular docking and further extraction-purification of the identified phytochemicals for sub-clinical and clinical trials, considering several putative mechanisms underlying these associations may be offered. Most obesity-treatment drugs are appetite suppressants; however, such agents have a number of limitations, such as side effects and a high recurrence rate. Therefore, the use of agricultural products is recommended. Moreover, applying production strategies will increase the functionality of the product. For RI, it is important to recognize the appropriate plant development stage to start the hydric stress.

**Figure 6:**
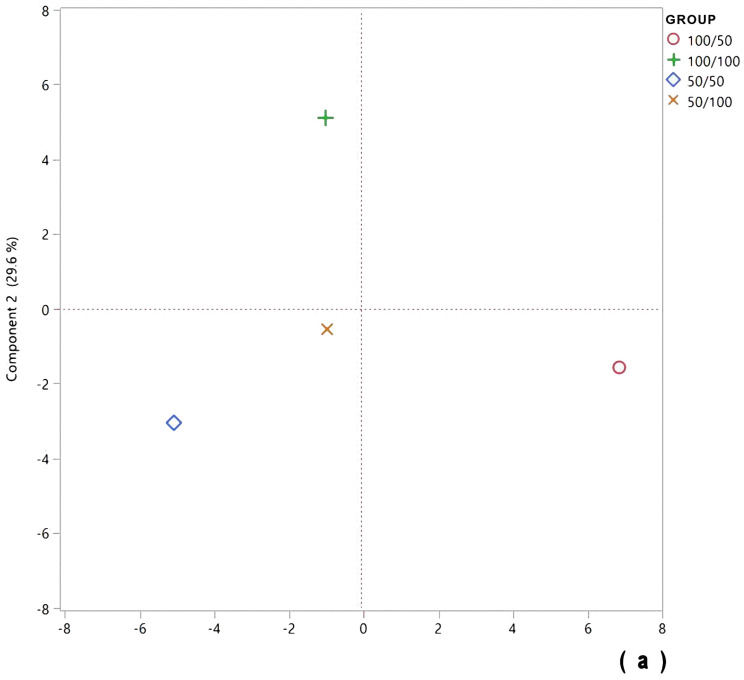

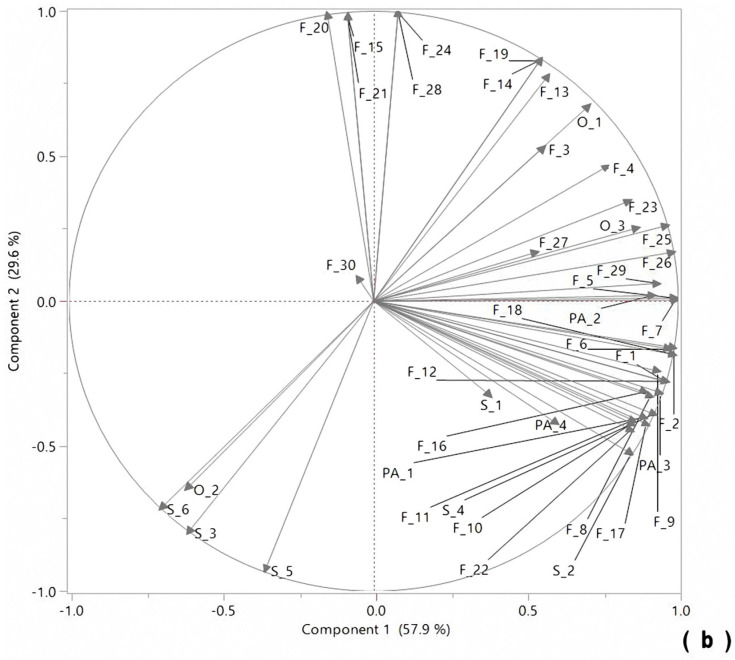
Principal component analysis for the seed phytochemicals of cooked Dalia harvested from plants grown under different levels of soil moisture during the growing cycle. Score plots (**a**) and loading plot (**b**). Bean seeds obtained by 100/50-treated plants had the greatest concentrations of a high number of phytochemicals, as this sample is placed in the right lower quadrant of the score plot. In analyzing the loading plot, a high number of phytochemicals are placed in the same quadrant.

In accordance, we performed a principal component analysis to identify which of the targeted metabolites were highlighted by the effect of the RI. PC1 and PC2 explained almost 88% of the total variation (Figure 6). The score plot shows that the Dalia plants cultivated under RI led to a higher synthesis of several metabolites (Figure 6a), displayed as higher positive values in the PC1–located in the right lower quadrant of the loading plot (Figure 6b). From the previously identified phytochemicals, kaempferol acetyl-hexoside, kaempferol malonyl-hexoside, luteolin, quercetin hexoside-rhamnoside, kaemperol hexoside, (Iso)rhoifolin, soyasaponin Ba (V), and soyasaponin Bb (I) were highly synthetized by the 100/50 treatment. Conversely, the high negative values of PC1 accounted for a higher concentration of a few of the saponins (soyasaponins ag, bg, and gg) in the plants submitted to a RI during the vegetative stage (50/100) of plant development or the whole cycle (50/50). As previously mentioned, both treatments resulted in an improvement in the liver enzymes activity measured directly in the tissue.

## 3. Material and Methods

### 3.1. Dalia Bean Origin and Selection

Dalia was obtained from the single cross of Flor de Junio Silvia (FJS), a sister line of Flor de Junio Marcela X Flor de Mayo Anita (FMA). The cross was made in the Bajío Experimental Station (CEBAJ) of the National Institute of Forestry, Agriculture and Livestock Research (INIFAP), under irrigated conditions in 2003. The selection of Dalia was made in the CEBAJ, under irrigation and rainfed conditions in two alternate generations per year. Within the early generation F2, and the intermediate F6, the selection was based on the health of plants, number of pods, and grain characteristics. From the generation F3, Dalia selection was between and within the families, and in F8, was considered phenotypically uniform. For this reason, it was joined with other 34 F8 lines in a preliminary yield trial during the spring–summer, 2006 cycle. Dalia is of indeterminate-prostrates habit, type 3 from the Jalisco race and has grains of cream-colored background with pink stripes [30]. This cultivar was registered at the National Seed Inspection and Certification Service (SNICS) with registration number FRI-077-240512, obtained from the National Catalog of Plant Varieties [31]. The obtainment of Dalia seed for the current experimental work was during the 2016 spring-summer cycle in the Zacatecas Research Station-INIFAP. The Dalia seed is sheltered and cultivated at INIFAP for its subsequent donation, exchange, and analysis.

### 3.2. Experimental Design Followed for Bean Production

**Figure 7:**
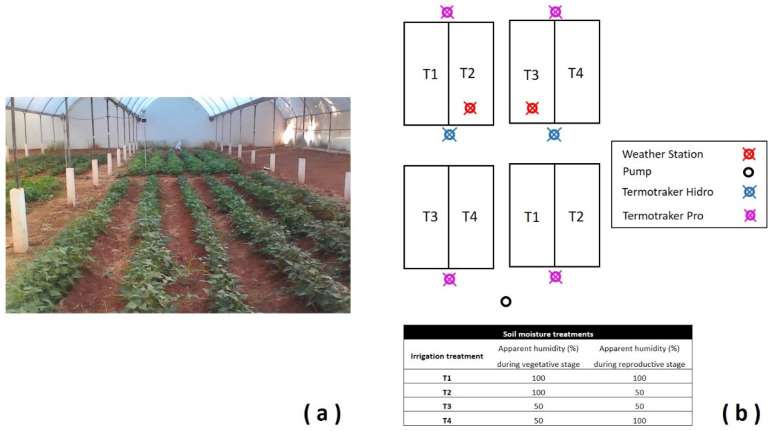
Experimental plot with common bean cv. Dalia under a rainout shelter (**a**) and sketch of the plot (**b**).

The experimental plot for bean production is depicted in Figure 7a. An experimental randomized complete block design was followed. Each block was replicated four times and comprised six rows of which 5 m of linear length were sown with Flor de Junio Dalia. Subsequently, all plots were sub-divided at the ending of the vegetative stage for establishment of the irrigation treatment. Plastic sheets 0.5 m long were placed into the ground to separate the sub-divisions at the beginning of the reproductive stage. A drip irrigation system was installed using ½ inch diameter polyethylene drip tubing, with emitters at 0.20 m and a water flow rate of 0.94 L/h per emitter. Soil moisture was evaluated using a Watermark^®^ humidity sensor (200SS-5, Irrometer, Riverside, CA, USA) installed in each experimental plot at 30 cm of depth. Measurements were done daily. Soil moisture was obtained in centibars/kilopascals. Treatments were 100/100, 100/50, 50/50, and 50/100 of soil moisture during vegetative/reproductive stage (Figure 7b). Plants with signs of aerial or root disease symptoms were removed from the experiment, as well as those from each end of the rows (0.5 m). At physiological maturity, plants were hand harvested and threshed.

### 3.3. Sample Preparation and Phytochemicals Quantification

After threshing, bean grains were washed in clean water, cooked (1:5, *w*/*v* at 90 ± 1 °C), and freeze-dried (Labconco, Kansas City, MO, USA) for at least 10 days, which is mandatory for a moisture content less than 0.5% [10]. Thereafter, samples were milled (Krups GX4100, Solingen, Germany, and stored in plastic bags.

For bean extraction, 100 mg were mixed with 1 mL of MS-grade methanol in an ultrasonic bath at 40 KHz (08895-75, Cole-Parmer, Vernon Hills, IL, USA) for 30 min. Then, samples were centrifuged at 16,000× *g* for 10 min at 4 °C. Supernatants were recovered and the extraction was repeated with the pellet. Both supernatants were mixed and concentrated in a vacuum centrifuge (SC210A, Thermo Scientific, Waltham, MA, USA) for 12 h. Samples were resuspended in 200 µL of MS-grade methanol, filtered using a PVDF syringe filter (13 mm, 0.45 µm), and stored in ambar vials at −20 °C.

The phytochemical profile was assessed in an Ultra-Performance Liquid Chromatograph (UPLC) coupled to a Diode Array Detector and a Quadrupole Time-of-Flight mass spectrometer with an electrospray ionization interphase (Vion IMS, Waters Co., Milford, MA, USA). Samples were filtered (0.45 mm) and directly injected into a BEH Acquity C18 column (2.1 × 100 mm, 1.7 mm) at 35 °C. Conditions for UPLC analysis were previously reported by Reynoso-Camacho et al. [32]. Commercial standards were used for quantification († in results Table 1). Data acquisition were performed using high definition MSE positive and negative ionization mode with a mass range of 50–2000 Da with UNIFI Scientific Information System (Waters Co., Milford, MA, USA). Peak identity was established by analyzing their exact mass (confirmation of elemental composition with <5 ppm mass error) and characteristic fragments. Quantification was carried out with calibration curves using available commercial standards.

### 3.4. Experimental Animals

The experimental design followed for in vivo tests was previously described by Salas-Lumbreras et al. [22] (Supplementary 1). The work reports response variables such as body weight, lipid profile, atherosclerosis index, glucose, and insulin resistance in the same batch of experimental animals (UAZ-2015–36851) subjected to the current research work. Bean samples from all soil moisture treatments showed a similar nutritional composition: protein, 19.7–21.2; lipids, 1.8–2.3; ash, 3.7–4.4; and carbohydrates: 72.8–74.4 g/100 g.

Young male Wistar rats with a body weight of 70–110 g (Universidad Autónoma de Zacatecas, Mexico City, Mexico) were used in the experiment (UAZ-2015-36851). Experimental animals were maintained at a controlled temperature (25 ± 1 °C) and humidity (55 ± 15%) under a 12-h light/dark cycle, housed in acrylic cages (32 × 47 × 20) with stainless steel wire grid lids (two to three animals per cage), with a total of 25 cages. Animal care was done by the same bioterium staff to ensure that all animals in the experiment were handled, monitored, and treated the same way. Food and water were provided ad libitum. Experiments were performed in accordance with the Mexican guidelines (NOM-062-ZOO-1999) and the National Institute of Health (National Institutes of Health, 2002) recommendations for animal research, prior authorization of the protocol by the Committee of the Universidad Autónoma de Zacatecas. All experiments were performed in accordance with relevant guidelines and regulations.

After one week of adaptation, the Wistar rats were assigned into 6 groups at random (n = 10 per group): a healthy control group was fed a standard diet of 3.39 calories per gram of food (12% moisture, 23% protein, 3% lipids, 7% ash, 49% carbohydrates and 6% total fiber) and obese groups fed a high fat diet (HFD). The HFD was prepared by adding 32% lean fat to the standard diet resulting in an intake of 6.27 calories per gram of food. After four months of follow-up, the HFD-feed animals were reassigned, into an obese control, and four treated-groups with cooked common bean. Treated-groups were assigned according to the water supplementation regimens during the vegetative and reproductive stage of Dalia production (100/100, 100/50, 50/50, and 50/100 vegetative/reproductive stage), each of these groups were supplemented with 20% (p/p) of cooked beans obtained from the different Dalia bean plants. Food and water were provided ad libitum. After three more months of treatment with cooked Dalia beans, the animals were sacrificed under anesthesia to obtain a blood sample by cardiac puncture and obtain the serum that was immediately separated and stored at −80 °C until analysis. The sacrifice of animals was performed by a technician of the bioterium for concealment and blinding experiments. Each response variable was evaluated and analyzed by a team member involved in the current experimental work. The reported experiments in the manuscript follow the recommendations in the ARRIVE guidelines.

### 3.5. Quantification of Liver Enzymes Activity

The levels of AST, ALT, GGT, and APT activities were measured in serum samples using commercial kits (BioSystems S.A., Barcelona, Spain). In addition, liver extracts were obtained by adding 1 mL of phosphate buffer to 100 mg of tissue and homogenate at 5000 RPM using a disperser (IKA T18 basic. Ultra-Turrax, Wilmington, NC, USA) placing tubes into a water bath. Enzyme activity was also measured.

## 4. Statistical Analysis

Data were expressed as mean ± the standard error (SEM). The statistical analysis was performed was an analysis of variance (ANOVA). Significant differences were considered when *p* ≤ 0.05 with the Tukey’s test. Plots were constructed using the GraphPad Prism 8.0.1 software. A supervised partial least square discriminant analysis (PLS-DA) was used to determine the relationship between the Dalia phytochemicals obtained by different water regimes and the resulting activities of liver enzymes were assessed. Plots were constructed with the variable importance of projection (VIP) against coefficient scores using non-linear iterative partial least squares (NIPALS). A principal component analysis (PCA) was also carried out. Analyses were conducted using JMP software V.5.0.1.

## 5. Conclusions

Since RI has been widely researched as the application of water below full crop water requirements during drought-sensitive growth stages, identifying a better water supply regime to improve food quality and functionality is of major concern. On the other hand, several studies have researched the functional properties of a variety of common bean genotypes which found that the Dalia cultivar has been proposed as an outstanding genotype because it is rich in flavonoids and has the capacity to improve the lipids profile, inflammatory citokines, the expression of lipid and carbohydrate metabolism genes, and insulin resistance. However, the latter responses associated with Dalia intake have been obtained from experiments conducted without considering the agroclimatic factors acting during plant growth and development. To the best of our knowledge, this is one of the few studies where the production of the common bean under different soil moisture scenarios is done with the purpose of enhancing the functionality of a well-known high-quality bean genotype. RI during the reproductive stage of the common bean plants’ development improved the phytochemical fingerprint of the harvested beans without penalizing the seed yield. The application of multivariate statistical analyses, such as chemometric tools (PLS-DA and PCA), led to the identification of RI-induced phytochemicals with a higher capacity to counteract abnormal liver functions and revealed that the RI during the reproductive stage of bean growth contributed to the enhanced synthesis of important phytochemicals.

## Figures and Tables

**Table 1:** Phytochemical fingerprint of Dalia bean seeds from plants grown under different soil moisture treatments.

Family	Code	Component Name	Retention Time (min)	Molecular Formula	Expected Mass (Da)	Observed Mass (Da)	Observed *m*/*z* ††	Mass Error (ppm)	Soil Moisture Treatments				*p* Value
									100/100	100/50	50/50	50/100	
Flavanols	F_1	(−)-Epicatechin hexoside	2.05	C_21_H_24_O_11_	452.1319	452.1332	451.1259	3.00	15.10 ± 1.78 b	31.75 ± 4.15 a	14.13 ± 1.71 b	17.71 ± 2.19 b	0.0087
	F_2	(+)-Catechin hexoside	2.61	C_21_H_24_O_11_	452.1319	452.1330	451.1257	2.50	4.52 ± 0.58 ab	7.24 ± 0.93 a	3.84 ± 0.48 b	5.15 ± 0.67 ab	0.0288
	F_3	(+)-Catechin †	2.62	C_15_H_14_O_6_	290.0790	290.0798	289.0725	2.56	14.73 ± 1.15 a	14.62 ± 1.16 a	12.93 ± 1.01 a	15.07 ± 1.23 a	0.3625
	F_4	(−)-Epicatechin †	3.03	C_15_H_14_O_6_	290.0790	290.0798	289.0726	2.75	5.76 ± 0.38 a	5.91 ± 0.44 a	4.95 ± 0.34 a	5.81 ± 0.44 a	0.2036
	F_5	Procyanidin dimer B2 †	3.12	C_30_H_26_O_12_	578.1424	578.1434	577.1361	1.67	0.66 ± 0.16 a	1.20 ± 0.36 a	0.36 ± 0.10 a	0.76 ± 0.22 a	0.0868
Flavonols	F_6	Quercetin hexoside-rhamnoside	3.87	C_27_H_30_O_16_	610.1534	610.1537	609.1464	0.56	0.63 ± 0.13 a	0.94 ± 0.19 a	0.57 ± 0.12 a	0.62 ± 0.13 a	0.1812
	F_7	Quercetin hexoside	4.26	C_21_H_20_O_12_	464.0955	464.0964	463.0891	1.90	3.78 ± 0.07 b	5.30 ± 0.62 b	3.03 ± 0.01 b	3.53 ± 0.07 a	0.0076
	F_8	Kaempferol pentosyl-hexoside	4.30	C_26_H_28_O_15_	580.1428	580.1434	579.1361	0.96	4.47 ± 0.04 b	10.04 ± 0.44 a	4.73 ± 0.03 b	4.59 ± 0.00 b	<0.0001
	F_9	Kaempferol hexoside	5.26	C_21_H_20_O_11_	448.1006	448.1015	447.0942	2.00	217.70 ± 2.18 b	256.42 ± 4.95 a	215.44 ± 2.79 b	215.25 ± 2.38 b	0.0006
	F_10	Kaempferol acetyl-hexoside	6.28	C_23_H_22_O_12_	490.1111	490.1116	489.1043	0.97	3.81 ± 0.48 b	10.26 ± 1.33 a	4.97 ± 0.64 b	4.06 ± 0.54 b	0.0041
	F_11	Kaempferol malonyl-hexoside	6.33	C_24_H_22_O_14_	534.1010	534.1008	533.0935	−0.36	0.30 ± 0.04 b	0.65 ± 0.14 a	0.36 ± 0.05 ab	0.31 ± 0.07 ab	0.0408
	F_12	Quercetin rhamnoside	6.52	C_21_H_20_O_11_	448.1006	448.1013	447.0940	1.68	6.43 ± 0.70 a	9.12 ± 1.02 a	6.27 ± 0.68 a	6.86 ± 0.72 a	0.0649
	F_13	Quercetin acetyl-rhamnoside	8.84	C_23_H_22_O_12_	490.1111	490.1108	489.1035	−0.69	0.24 ± 0.03 a	0.21 ± 0.01ab	0.13 ± 0.00 b	0.20 ± 0.04 ab	0.0460
	F_14	Quercetin †	8.99	C_15_H_10_O_7_	302.0427	302.0424	301.0351	−0.98	0.13 ± 0.02 a	0.11 ± 0.01 ab	0.07 ± 0.01 b	0.09 ± 0.01 ab	0.0290
	F_15	Kaempferol †	11.12	C_15_H_10_O_6_	286.0477	286.0469	285.0396	−3.03	17.55 ± 1.51 a	7.34 ± 0.57 b	6.56 ± 0.50 b	8.04 ± 0.63 b	0.0008
Flavones	F_16	Luteolin apiosyl-hexoside	4.30	C_26_H_28_O_15_	580.1428	580.1434	579.1361	0.96	4.53 ± 0.00 c	9.48 ± 0.10 a	4.80 ± 0.03 b	4.23 ± 0.00 d	<0.0001
	F_17	Luteolin †	6.25	C_15_H_10_O_6_	286.0477	286.0479	285.0406	0.65	0.07 ± 0.01 b	0.14 ± 0.02 a	0.08 ± 0.01 b	0.08 ± 0.01 b	0.0070
	F_18	(Iso)rhoifolin	8.85	C_27_H_30_O_14_	578.1636	578.1614	577.1541	−3.72	0.12 ± 0.03 a	0.19 ± 0.03 a	0.10 ± 0.02 a	0.13 ± 0.02 a	0.1160
	F_19	Hydroxyluteolin	8.99	C_15_H_10_O_7_	302.0427	302.0424	301.0351	−0.98	0.12 ± 0.01 a	0.10 ± 0.01 ab	0.06 ± 0.01 b	0.08 ± 0.01 ab	0.0290
	F_20	Sinensetin	11.02	C_20_H_20_O_7_	372.1209	372.1208	371.1135	−0.34	2.34 ± 0.25 a	0.37 ± 0.05 c	0.31 ± 0.04 c	0.96 ± 0.11 b	0.0004
	F_21	Scutellarein	11.12	C_15_H_10_O_6_	286.0477	286.0469	285.0396	−3.03	16.18 ± 1.39 a	6.77 ± 0.53 b	6.05 ± 0.46 b	7.41 ± 0.58 b	0.0008
Isoflavones	F_22	Genistein malonyl-hexoside (malonylgenistin)	3.49	C_24_H_22_O_13_	518.1060	518.1051	517.0978	−1.81	1.37 ± 0.18 a	2.15 ± 0.29 a	1.45 ± 0.19 a	1.83 ± 0.25 a	0.0853
	F_23	Daidzein hexoside (daidzin)	3.98	C_21_H_20_O_9_	416.1107	416.1124	415.1051	3.91	0.03 ± 0.00 a	0.05 ± 0.01 a	ND	ND	0.0010
	F_24	Tetrahydroxyisoflavanone	5.51	C_15_H_12_O_6_	288.0634	288.0636	287.0563	0.80	6.67 ± 0.46 a	4.24 ± 0.32 b	3.42 ± 0.25 b	4.35 ± 0.30 b	0.0027
	F_25	Genistein †	5.75	C_15_H_10_O_5_	270.0528	270.0531	269.0458	1.06	0.11 ± 0.01 ab	0.13 ± 0.02 a	0.07 ± 0.01 b	0.09 ± 0.01 ab	0.0213
	F_26	Daidzein †	7.63	C_15_H_10_O_4_	254.0579	254.0574	253.0501	−2.12	0.15 ± 0.01 ab	0.20 ± 0.03 a	0.10 ± 0.01 b	0.14 ± 0.01 ab	0.0205
	F_27	Trihydroxyisoflavan	8.24	C_15_H_14_O_4_	258.0892	258.0888	257.0816	−1.44	0.06 ± 0.01 ab	0.08 ± 0.01 a	0.05 ± 0.00 b	ND	0.0006
Flavanones	F_28	Eriodictyol	5.52	C_15_H_12_O_6_	288.0634	288.0636	287.0563	0.80	6.45 ± 0.44 a	4.11 ± 0.31 b	3.31 ± 0.25 b	4.21 ± 0.29 b	0.0027
	F_29	Naringenin hexoside	5.75	C_21_H_22_O_10_	434.1213	434.1221	433.1148	1.77	0.25 ± 0.04 a	0.34 ± 0.06 a	0.19 ± 0.03 a	0.19 ± 0.04 a	0.0687
	F_30	Naringenin †	10.95	C_15_H_12_O_5_	272.0685	272.0676	271.0603	−3.36	0.25 ± 0.02 b	0.23 ± 0.02 b	0.20 ± 0.02 b	0.43 ± 0.04 a	0.0031
Hydroxybenzoic acids	PA_1	Vanillic acid †	1.79	C_8_H_8_O_4_	168.0423	168.0417	167.0344	−3.26	0.49 ± 0.03 c	0.78 ± 0.06 a	0.50 ± 0.04 b	0.63 ± 0.04 ab	0.0063
	PA_2	Dihydroxybenzoic acid	1.91	C_7_H_6_O_4_	154.0266	154.0263	153.0190	−2.04	0.29 ± 0.00 b	0.42 ± 0.02 a	0.21 ± 0.01 c	0.36 ± 0.02 a	0.0007
	PA_3	Hydroxybenzoic acid hexoside	2.10	C_13_H_16_O_8_	300.0845	300.0841	299.0768	−1.36	0.32 ± 0.02 a	0.79 ± 0.26 a	0.30 ± 0.02 a	0.49 ± 0.14 a	0.0889
Hydroxycinnamic acids	PA_4	Ferulic acid †	3.96	C_10_H_10_O_4_	194.0579	194.0579	193.0507	0.10	0.08 ± 0.01 b	0.12 ± 0.01 a	0.09 ± 0.01 b	0.13 ± 0.01 a	0.0072
Oligosaccharides	O_1	Raffinose †	0.58	C_18_H_32_O_16_	504.1690	504.1705	503.1632	2.84	1.98 ± 0.25 a	1.96 ± 0.23 a	1.74 ± 0.22 a	1.91 ± 0.27 a	0.7628
	O_2	Stachyose	1.27	C_24_H_42_O_21_	666.2219	666.2217	665.2144	−0.26	4.21 ± 0.65 a	4.31 ± 0.71 a	4.97 ± 0.73 a	4.99 ± 0.76 a	0.6086
	O_3	Verbascose	3.13	C_30_H_52_O_26_	828.2747	828.2707	827.2634	−4.79	0.11 ± 0.05 a	0.17 ± 0.07 a	ND	0.14 ± 0.08 a	0.1405
Saponins	S_1	Soyasaponin Ba (V)	11.54	C_48_H_78_O_19_	958.5137	958.5114	957.5041	−2.47	48.17 ± 1.95 a	51.13 ± 0.60 a	48.52 ± 1.87 a	53.40 ± 0.55 a	0.0575
	S_2	Soyasaponin Bb (I) †	11.58	C_48_H_78_O_18_	942.5188	942.5166	941.5093	−2.34	11.11 ± 3.75 a	12.78 ± 4.33 a	11.46 ± 3.77 a	11.85 ± 4.00 a	0.975
	S_3	Soyasaponin ag	11.68	C_54_H_84_O_22_	1084.5454	1084.5450	1083.5378	−0.36	18.21 ± 0.65 b	18.78 ± 0.07 b	20.69 ± 0.29 a	19.49 ± 0.15 ab	0.0098
	S_4	Soyasaponin Bd	11.71	C_48_H_76_O_19_	956.4981	956.4968	955.4895	−1.31	2.07 ± 0.55 a	5.66 ± 1.52 a	2.34 ± 0.61 a	2.92 ± 0.79 a	0.0578
	S_5	Soyasaponin bg	11.74	C_54_H_84_O_21_	1068.5505	1068.5499	1067.5427	−0.54	3.16 ± 0.96 a	3.69 ± 1.48 a	4.27 ± 1.26 a	3.71 ± 1.12 a	0.8414
	S_6	Soyasaponin gg	11.80	C_48_H_74_O_17_	922.4926	922.4919	921.4846	−0.80	2.30 ± 0.97 a	2.35 ± 1.00 a	2.84 ± 1.02 a	2.53 ± 1.02 a	0.9443

Mean ± standard deviation (SD). Results are expressed as µg/g. Different letters in same row indicate significant differences (*p* < 0.05) by Tukey’s test. O: oligosaccharide; S: saponins; ND: not detected. † Identification confirmed with commercial standards. †† All molecular ions where identified at negative ionization mode as [M − H]^−^.

## Data Availability

All data generated and analyzed during this study are included in this published article and its supplementary information files. The datasets used and/or analyzed during the current study are available from the corresponding author on reasonable request.

## Supplementary Materials

The following supporting information can be downloaded at: https://www.mdpi.com/article/10.3390/molecules28247983/s1, Table S1. Variable Importance in Projection (VIP) scores. Table S2. *p* values for liver enzymes activity evaluated in serum. Table S3. *p* values for liver enzymes activity evaluated in hepatic tissue.
