# Supplementary material for: Chemometric Evaluation of RI-Induced Phytochemicals in Phaseolus vulgaris Seeds Indicate an Improvement on Liver Enzymes in Obese Rats
Source: Molecules. 2023 Dec 7;28(24):7983. doi: 10.3390/molecules28247983 (PMC10746056; doi:10.3390/molecules28247983)
Supplement: Supplementary file 1 [file molecules-28-07983-s001.zip › molecules-2706333-supplementary.pdf]

Chemometric Evaluation of RI-Induced Phytochemicals in *Phaseolus vulgaris* Seeds Indicate an Improvement on Liver Enzymes in Obese Rats

Mayra Denise Herrera [1,2], Iza Fernanda Pérez-Ramírez [3], Rosalía Reynoso-Camacho [3], Luis Roberto Reveles-Torres [1,2], Miguel Servín-Palestina [1], Angelica Judith Granados-López [2], Claudia Araceli Reyes-Estrada [4,*] and Jesús Adrián López [2,*]

[1] Campo Experimental Zacatecas (CEZAC-INIFAP), Carretera Zacatecas-Fresnillo Km 24.5, Calera de VR, Zacatecas 98500, Mexico; mayradherrera@gmail.com (M.D.H.); lreveles@gmail.com (L.R.R.-T.); servin.miguel@inifap.gob.mx (M.S.-P.)
[2] Unidad Académica de Ciencias Biológicas, Universidad Autónoma de Zacatecas "Francisco García Salinas", Avenida Preparatoria No. 301, Colonia Hidráulica, Zacatecas 98068, Mexico; agranados@uaz.edu.mx
[3] Research and Graduate Studies in Food Science, Faculty of Chemistry, Autonomous University of Queretaro, Queretaro 76010, Mexico; iza.perez@uaq.mx (I.F.P.-R.); rrcamachomx@yahoo.com.mx (R.R.-C.)
[4] Unidad Académica de Ciencias Químicas, Universidad Autónoma de Zacatecas, Campus Siglo XXI, Villanueva–Zacatecas, La Escondida, Zacatecas 98160, Mexico
* Correspondence: c_reyes13@uaz.edu.mx (C.A.R.-E.); jalopez@uaz.edu.mx (J.A.L.)

Table S1. Variable Importance in Projection (VIP) scores.

Phytochemical	Serum		Liver	
	AST, ALT	GGT, ALP	AST, ALT	GGT, ALP
F_1	1.152	1.057	0.343	0.831
F_2	1.045	1.060	0.534	0.794
F_3	0.497	0.840	1.086	0.772
F_4	0.533	0.909	1.061	0.903
F_5	0.893	1.080	0.699	0.835
F_6	1.060	1.155	0.474	0.920
F_7	0.932	1.190	0.679	0.986
F_8	1.175	1.091	0.259	0.878
F_9	1.108	1.149	0.395	0.912
F_10	1.227	1.042	0.156	0.866
F_11	1.217	1.054	0.181	0.869
F_12	1.151	1.057	0.346	0.830
F_13	0.784	0.715	1.246	1.205
F_14	0.871	0.893	1.463	1.384
F_15	1.326	0.482	1.374	1.246
F_16	1.148	1.127	0.328	0.897
F_17	1.255	0.988	0.165	0.827
F_18	1.076	1.080	0.466	0.835
F_19	0.871	0.893	1.463	1.384
F_20	1.372	0.194	1.174	1.189
F_21	1.326	0.482	1.374	1.246
F_22	1.204	0.905	0.745	0.633

F_23	0.670	1.341	1.237	1.241
F_24	1.247	0.445	1.384	1.306
F_25	0.747	1.189	0.964	1.112
F_26	0.786	1.113	0.833	0.967
F_27	0.503	1.463	1.365	1.026
F_28	1.247	0.445	1.384	1.306
F_29	0.859	1.295	0.855	1.078
F_30	0.213	1.324	1.319	0.527
PA_1	1.199	0.927	0.628	0.664
PA_2	0.793	1.024	0.838	0.646
PA_3	1.157	0.984	0.506	0.719
PA_4	0.950	1.040	1.252	0.475
O_1	0.678	0.853	1.214	1.181
O_2	0.702	1.255	1.551	1.364
O_3	0.600	0.993	0.956	0.763
S_1	0.653	1.197	1.437	0.452
S_2	1.312	0.856	0.524	0.745
S_3	0.815	0.854	1.375	1.333
S_4	1.234	0.986	0.275	0.800
S_5	1.028	0.544	1.340	1.306
S_6	0.728	0.905	1.301	1.279

VIP scores were obtained for hepatocellular (AST, ALT) and hepatobiliary (GGT, ALP) function separately for serum and liver samples.

Table S2. *p* values for liver enzymes activity evaluated in serum

**Tukey's multiple comparisons test**	**Adjusted P Value**	**Tukey's multiple comparisons test**	**Adjusted P Value**
**Serum AST**		**Serum GGT**	
Healthy ctrol vs. Obese ctrol	<0.0001	Healthy ctrol vs. Obese ctrol	<0.0001
Healthy ctrol vs. 100/100	<0.0001	Healthy ctrol vs. 100/100	0.4671
Healthy ctrol vs. 100/50	0.0978	Healthy ctrol vs. 100/50	0.8685
Healthy ctrol vs. 50/50	0.0335	Healthy ctrol vs. 50/50	0.0327
Healthy ctrol vs. 50/100	0.012	Healthy ctrol vs. 50/100	0.0082
Obese ctrol vs. 100/100	0.9997	Obese ctrol vs. 100/100	0.0019
Obese ctrol vs. 100/50	0.0092	Obese ctrol vs. 100/50	0.0003
Obese ctrol vs. 50/50	0.0302	Obese ctrol vs. 50/50	0.0622
Obese ctrol vs. 50/100	0.0783	Obese ctrol vs. 50/100	0.1941
100/100 vs. 100/50	0.0188	100/100 vs. 100/50	0.9804
100/100 vs. 50/50	0.0582	100/100 vs. 50/50	0.7255
100/100 vs. 50/100	0.1408	100/100 vs. 50/100	0.3861

100/50 vs. 50/50	0.9965	100/50 vs. 50/50	0.3128
100/50 vs. 50/100	0.9432	100/50 vs. 50/100	0.1129
50/50 vs. 50/100	0.9981	50/50 vs. 50/100	0.9928
**Serum ALT**		**Serum GGT**	
Healthy ctrol vs. Obese ctrol	<0.0001	Healthy ctrol vs. Obese ctrol	<0.0001
Healthy ctrol vs. 100/100	0.0002	Healthy ctrol vs. 100/100	0.0042
Healthy ctrol vs. 100/50	0.1031	Healthy ctrol vs. 100/50	0.0396
Healthy ctrol vs. 50/50	0.0019	Healthy ctrol vs. 50/50	0.0002
Healthy ctrol vs. 50/100	0.0014	Healthy ctrol vs. 50/100	0.0015
Obese ctrol vs. 100/100	0.9561	Obese ctrol vs. 100/100	0.4308
Obese ctrol vs. 100/50	0.0255	Obese ctrol vs. 100/50	0.0876
Obese ctrol vs. 50/50	0.5468	Obese ctrol vs. 50/50	0.9612
Obese ctrol vs. 50/100	0.6174	Obese ctrol vs. 50/100	0.6667
100/100 vs. 100/50	0.1614	100/100 vs. 100/50	0.943
100/100 vs. 50/50	0.9561	100/100 vs. 50/50	0.8921
100/100 vs. 50/100	0.9761	100/100 vs. 50/100	0.9988
100/50 vs. 50/50	0.5821	100/50 vs. 50/50	0.3852
100/50 vs. 50/100	0.5118	100/50 vs. 50/100	0.7902
50/50 vs. 50/100	>0.9999	50/50 vs. 50/100	0.983

Table S3. *p* values for liver enzymes activity evaluated in hepatic tissue

**Tukey's multiple comparisons test**	**Adjusted P Value**	**Tukey's multiple comparisons test**	**Adjusted P Value**
**Liver AST**		**Liver GGT**	
Healthy ctrol vs. Obese ctrol	<0.0001	Healthy ctrol vs. Obese ctrol	0.042
Healthy ctrol vs. 100/100	0.0962	Healthy ctrol vs. 100/100	0.3391
Healthy ctrol vs. 100/50	0.8953	Healthy ctrol vs. 100/50	0.972
Healthy ctrol vs. 50/50	0.9803	Healthy ctrol vs. 50/50	0.1337
Healthy ctrol vs. 50/100	0.9992	Healthy ctrol vs. 50/100	0.9875
Obese ctrol vs. 100/100	0.0739	Obese ctrol vs. 100/100	0.909
Obese ctrol vs. 100/50	0.0008	Obese ctrol vs. 100/50	0.0054
Obese ctrol vs. 50/50	<0.0001	Obese ctrol vs. 50/50	<0.0001
Obese ctrol vs. 50/100	<0.0001	Obese ctrol vs. 50/100	0.0078
100/100 vs. 100/50	0.5748	100/100 vs. 100/50	0.0768
100/100 vs. 50/50	0.0171	100/100 vs. 50/50	0.0005
100/100 vs. 50/100	0.1979	100/100 vs. 50/100	0.102

100/50 vs. 50/50	0.5004	100/50 vs. 50/50	0.4872
100/50 vs. 50/100	0.9803	100/50 vs. 50/100	>0.9999
50/50 vs. 50/100	0.8953	50/50 vs. 50/100	0.4102
**Liver ALT**		**Liver GGT**	
Healthy ctrol vs. Obese ctrol	0.0093	Healthy ctrol vs. Obese ctrol	<0.0001
Healthy ctrol vs. 100/100	0.2943	Healthy ctrol vs. 100/100	0.4446
Healthy ctrol vs. 100/50	0.5504	Healthy ctrol vs. 100/50	0.0312
Healthy ctrol vs. 50/50	0.7677	Healthy ctrol vs. 50/50	0.2984
Healthy ctrol vs. 50/100	>0.9999	Healthy ctrol vs. 50/100	0.0296
Obese ctrol vs. 100/100	0.6626	Obese ctrol vs. 100/100	<0.0001
Obese ctrol vs. 100/50	0.3883	Obese ctrol vs. 100/50	<0.0001
Obese ctrol vs. 50/50	0.2158	Obese ctrol vs. 50/50	0.0445
Obese ctrol vs. 50/100	0.0093	Obese ctrol vs. 50/100	0.385
100/100 vs. 100/50	0.9977	100/100 vs. 100/50	0.7706
100/100 vs. 50/50	0.9665	100/100 vs. 50/50	0.0035
100/100 vs. 50/100	0.2943	100/100 vs. 50/100	0.0001
100/50 vs. 50/50	0.9992	100/50 vs. 50/50	<0.0001
100/50 vs. 50/100	0.5504	100/50 vs. 50/100	<0.0001
50/50 vs. 50/100	0.7677	50/50 vs. 50/100	0.887
